# Cofilin: a redox sensitive mediator of actin dynamics during T-cell activation and migration

**DOI:** 10.1111/imr.12115

**Published:** 2013-10-10

**Authors:** Yvonne Samstag, Isabel John, Guido H Wabnitz

**Affiliations:** 1 Institute for Immunology, Ruprecht-Karls-UniversityHeidelberg, Germany

**Keywords:** costimulation, T-cell activation, immune synapse, microenvironment, redox, actin cytoskeleton

## Abstract

Cofilin is an actin-binding protein that depolymerizes and/or severs actin filaments. This dual function of cofilin makes it one of the major regulators of actin dynamics important for T-cell activation and migration. The activity of cofilin is spatio-temporally regulated. Its main control mechanisms comprise a molecular toolbox of phospho-, phospholipid, and redox regulation. Phosphorylated cofilin is inactive and represents the dominant cofilin fraction in the cytoplasm of resting human T cells. A fraction of dephosphorylated cofilin is kept inactive at the plasma membrane by binding to phosphatidylinositol 4,5-bisphosphate. Costimulation via the T-cell receptor/CD3 complex (signal 1) together with accessory receptors (signal 2) or triggering through the chemokine SDF1α (stromal cell-derived factor 1α) induce Ras-dependent dephosphorylation of cofilin, which is important for immune synapse formation, T-cell activation, and T-cell migration. Recently, it became evident that cofilin is also highly sensitive for microenvironmental changes, particularly for alterations in the redox milieu. Cofilin is inactivated by oxidation, provoking T-cell hyporesponsiveness or necrotic-like programmed cell death. In contrast, in a reducing environment, even phosphatidylinositol 4,5-bisphosphate -bound cofilin becomes active, leading to actin dynamics in the vicinity of the plasma membrane. In addition to the well-established three signals for T-cell activation, this microenvironmental control of cofilin delivers a modulating signal for T-cell-dependent immune reactions. This fourth modulating signal highly impacts both initial T-cell activation and the effector phase of T-cell-mediated immune responses.

## Introduction

T cells migrate through the body and communicate with other hematopoietic or tissue-resident cells. In each new environment, T cells must adapt to the prevalent micromilieu, and their surface receptors have to interact with ligands on other cells or the extracellular matrix. High flexibility of the entire cell body is a prerequisite for T-cell-mediated immune surveillance, including transition from the radially symmetric shape of unstimulated cells within the blood stream to bipolarly asymmetric cells during migration or immune synapse formation of T cells with antigen-presenting cells (APCs) or target cells 1. The actin cytoskeleton plays a central role for these shape changes. It provides a scaffold for protein clustering and signal transduction and serves as an engine that generates physical forces important for T-cell activation and migration 2–5. Thus, regulation of the actin cytoskeleton is pivotal for T-cell functions. The functional relevance of actin rearrangements is emphasized by the fact that they belong to the major energy-consuming processes in cells, and in nature, energy is seldom wasted 6–7. This review focuses on one essential protein that modulates the actin cytoskeletal architecture: the actin-severing and depolymerizing protein cofilin.

## Regulation of actin dynamics by cofilin

**Figure 1:**
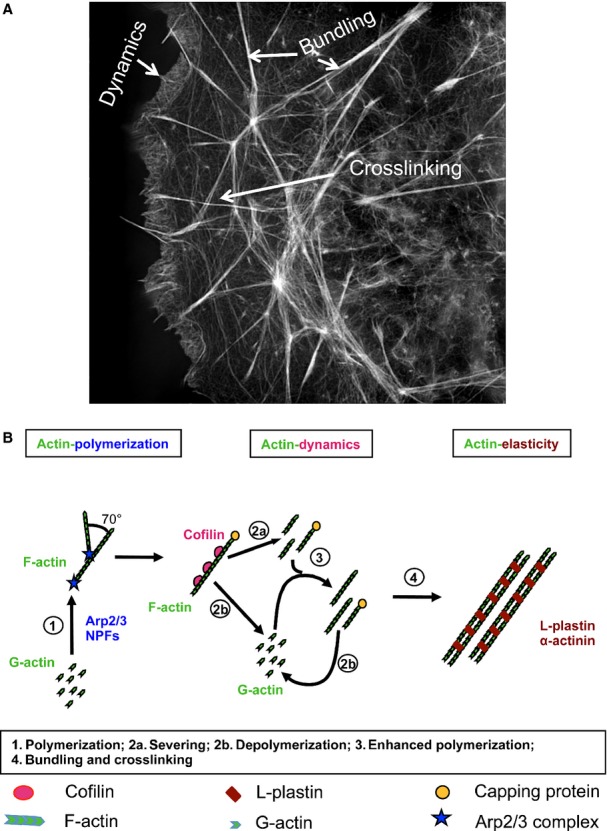
Structures and regulation of the actin cytoskeleton. (A) F-actin was stained in COS-7 cells with phalloidin (AlexaFluor-488) and acquired with structured-illumination microscopy (3D-SIM acquired in the SBIC-Nikon Imaging Centre at Biopolis, Heidelberg, Germany). The most dynamic actin reorganization takes place at the migratory front, although actin dynamics are important throughout the whole cell. F-actin bundling or crosslinking mediates elasticity. (B) Actin polymerization, dynamics, and elasticity. Key proteins for each step are depicted (reviewed in 2–5). Arp2/3, actin-related protein 2/3; NPFs, nucleation-promoting factors.

The actin cytoskeleton is a meshwork composed of 42 kDa globular units (G-actin) that can be reversibly polymerized to polar filaments (F-actin). Several nucleation-promoting factors, including the Wiskott-Aldrich syndrome protein (WASP), the WASP family Verprolin-homologous protein 2, and the hematopoietic lineage cell–specific protein 1, regulate the initiation of actin polymerization through activation of the actin-related protein 2/3 complex 8–14 (*Fig. *1). The pronounced phenotype of patients suffering from Wiskott-Aldrich syndrome and the knowledge about the relevance of WASP for actin polymerization 15–16 focused immunological research initially on WASP and other proteins important for actin polymerization, while actin-remodeling events other than actin polymerization were hardly considered for a long time. This changed during the last decade, during which time the importance of dynamic rearrangements of the actin cytoskeleton became evident (reviewed in 5,17). It is now known that actin reorganization is tightly controlled in a spatio-temporal fashion during T-cell activation. Proteins that bind two actin filaments, like L-plastin 19–24 or α-actinin 25,26, coordinate the complex actin meshwork through their crosslinking and bundling activity (*Fig. *1). Crosslinking or bundling of actin filaments causes an increase in cytoskeletal elasticity, thereby stabilizing the cell shape as well as cellular interactions. Notably, the actin cytoskeleton is not unidirectionally polymerized and/or crosslinked. Instead, it undergoes constant depolymerization/enhanced polymerization cycles termed ‘actin dynamics’. These are mediated primarily by cofilin 5–28 (*Fig. *1).

**Figure 2:**
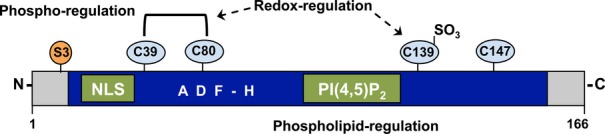
Regulation sites of cofilin. Cofilin is a highly regulated protein. The figure displays a threefold regulation of cofilin: phospho-regulation at serine 3, redox regulation at cysteine residues, and phospholipid regulation via PI(4,5)P_2_ binding.

Cofilin is a 19 kDa protein that is ubiquitously expressed in all mammalian cells. It contains an actin-depolymerizing factor homology (ADF-H) domain, which enables stoichiometric binding of cofilin to both G- and F-actin 29 (*Fig. *2). Binding of cofilin to F-actin is only weakly influenced by temperature but is highly dependent on salt concentration and pH value 30,31. Phosphorylation on serine 3 inactivates cofilin 33,34 by generation of a charge repulsion between cofilin and actin, which is thought to occur without altering the protein structure 36. Cofilin is evolutionarily highly conserved and belongs to the ADF/cofilin family of actin-binding proteins. The family consists of three isoforms: non-muscle cofilin [n-cofilin or cofilin-1 (CFL-1)], muscle-specific cofilin [m-cofilin or cofilin-2 (CFL-2)], and ADF (or destrin) 28. This review focuses on cofilin-1, which is highly expressed in human T cells 37.

Cofilin disassembles actin filaments by increasing the off-rate of ADP-actin at the so-called minus-pole (or pointed-end) of actin filaments. Thereby, cofilin depolymerizes F-actin on the one hand and frees ADP-actin, making it available for recycling to ATP-actin on the other hand. In addition, cofilin is able to sever F-actin, which shortens the overall length of the remaining actin filaments 38. Despite the fact that severing of F-actin into smaller pieces is one mechanism of F-actin disassembly, it also multiplies the number of actin filaments. Each of those F-actin fragments is prone to grow by polymerization at the barbed-end, which induces a net enhanced actin polymerization if the barbed-ends are not covered by capping proteins 5–38. Notably, although actin rearrangements demand a high supply of energy, mere actin severing by cofilin is independent of energy addition, as for example generated by ATP hydrolysis. This makes enhanced actin polymerization to an energetically favorable molecular mechanism compared to *de novo* actin nucleation 30. Whether cofilin activity results in F-actin shrinking or enhanced polymerization depends on the conditions and availability of G-actin in the specific area within the cell 39,40 and is likely influenced by different signaling cascades.

The dual function of cofilin, namely depolymerization and severing, makes it a key molecule controlling actin dynamics. Therefore, it is not surprising that cofilin expression is essential for cell survival. Cofilin knockout mice exhibit an embryonic lethal phenotype 42, and cofilin null mutants are also lethal in yeast 43. Due to this essential role, cofilin needs to be tightly controlled. Both extrinsic factors of the microenvironment and intrinsic signal transduction events mediate this cofilin orchestration through phospho-, phospholipid, and redox regulation of cofilin within human T cells (*Fig. *2). The signal transduction pathways and the microenvironmental elements regulating cofilin are discussed below.

## Cofilin activation induced by T-cell costimulation

### Costimulation of human T cells leads to cofilin activation through dephosphorylation

T cells recognize antigens bound to major histocompatibility complex (MHC) molecules on APCs via their antigen-specific TCR/CD3 complex. For induction of clonal growth and development of full functionality, T cells require not only the competence signal through TCR/CD3 triggering but also costimulatory signals through accessory receptors (e.g. CD2 or CD28). Absence of costimulation is one of the mechanisms leading to clonal anergy and antigen-specific tolerance 44,45.

In our studies of signaling processes specifically occurring after stimulation of accessory receptors on untransformed human peripheral blood T cells, we uncovered an important role for cofilin 37–47. Our initial experimental model was T-cell activation through the ‘alternative pathway’ 48–49, where stimulation of human T cells with monoclonal antibodies directed against two or three different epitopes of the CD2 molecule is able to fully activate T cells, even in the absence of antigen and APCs. While many signal transduction mechanisms are similar in terms of whether T cells are stimulated via the TCR/CD3-complex or via the ‘alternative pathway’ of T-cell activation, two-dimensional gel-electrophoresis of cells loaded with radioactively labeled orthophosphate showed that cofilin (which is constitutively phosphorylated on serine 3 in the cytoplasm of resting untransformed T cells) undergoes dephosphorylation following CD2 stimulation but not following triggering of TCR/CD3 alone 37–47. The principal independence of this signaling process from triggering of the TCR on the cell surface was confirmed by use of a CD2^+^ human NK cell clone in which cofilin likewise undergoes dephosphorylation following CD2 stimulation 47.

Induction of cofilin dephosphorylation/activation through the accessory receptor CD2 but not through TCR/CD3 alone gave rise to the assumption that cofilin represents a mediator of T-cell costimulation. Indeed, this dephosphorylation event is observed if T-cell stimulation through TCR/CD3 is accomplished by stimulation through the accessory receptors CD2 or CD28 50,51. Accordingly, in untransformed human T cells, expression of the T-cell growth factor IL-2, which represents a hallmark of T-cell activation after costimulation, is drastically reduced, if the interaction of cofilin with actin is blocked by cell-permeable cofilin peptide homologues 53.

Similar to CD2 stimulation, an activation pathway via CD28 (in the absence of TCR/CD3 triggering) has been described. This pathway is inducible by ‘superagonistic’ CD28 antibodies 54. Note, however, that in contrast to ‘alternative pathway’ activation via sole stimulation of CD2, dephosphorylation and activation of cofilin does not occur if untransformed human T cells are activated through ‘superagonistic’ CD28 antibodies 52. The same holds true for phosphorylation of the actin-bundling protein L-plastin, another costimulation-related signaling event, which coordinates receptor polarization upon T-cell costimulation 19–55. Intriguingly, while CD3/CD28 costimulation induces polarized large receptor clusters (caps) in T cells, crosslinking of ‘superagonistic’ CD28 antibodies provokes only small receptor clusters that fail to coalesce at one pole of the cell. Thus, ‘superagonistic’ CD28 stimulation does not mimic T-cell costimulation. Instead it represents induction of a cofilin and L-plastin independent state of ‘unpolarized’ T-cell activation 52.

### Cofilin-dependent actin dynamics are crucial for the maturation of the immune synapse

**Figure 3:**
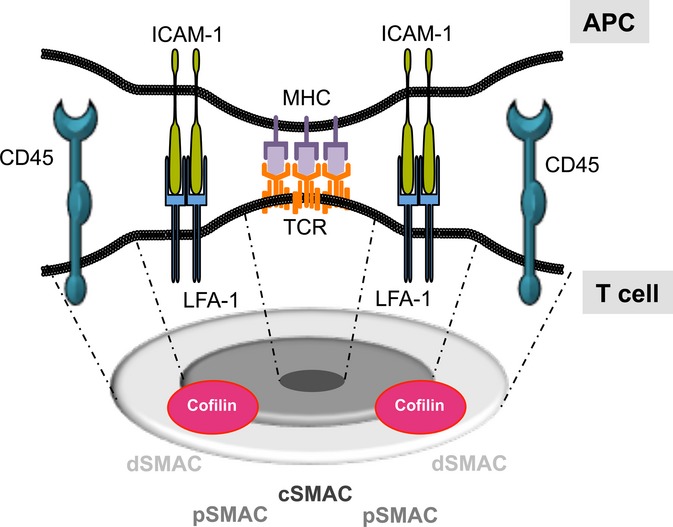
Cofilin is required for organized receptor clustering in the immune synapse. Upper part: Contact zone between APCs and T cells. Lower part: *En face* view of the bull's-eye shaped organization of the SMACs in the T-cell membrane. Cofilin localizes to the pSMAC and dSMAC. Immune synapses have been comprehensively reviewed 172,173.

Upon antigen recognition, a specialized contact zone between T cells and APCs is formed, in which these cells communicate and transduce signals leading to T-cell activation, proliferation, and differentiation. Analogous to the neuronal synapse, this contact zone is called immune synapse 56. Actin dynamics in the immune synapse induce radio symmetric forces from the edge inwards, and an organized receptor movement (as microclusters) in the immune synapse is generated 57–60. During this immune synapse maturation, surface receptors and cytoplasmic proteins are segregated in supramolecular activation clusters (SMACs) within the T-cell/APC contact zone. Three SMACs have been described, which are named central SMAC (cSMAC), peripheral SMAC (pSMAC), and distal SMAC (dSMAC), according to their relative localization in the cell interface (*Fig. *3). The cSMAC contains the TCR/CD3 complex, whereas the pSMAC has a high content of lymphocyte function-associated antigen 1 (LFA-1), and the dSMAC harbors receptors with large extracellular domains like CD45 (reviewed in 1–61). Notably, both costimulation and sustained actin dynamics are important to build and maintain a mature immune synapse 4–65. Upon antigen recognition, cofilin is enriched concentrically at the periphery of the contact zone, that is, the pSMAC and dSMAC 53–66 (*Fig.* 3). Interaction of cofilin with actin is crucial for receptor redistribution and actin dynamics within this contact zone 53. Moreover, the effects of cofilin for protein segregation at the immune synapse are receptor specific. Thus, inhibition of cofilin by cofilin-derived, cell-permeable peptides that interfere with the binding of cofilin to actin reduces accumulation of the costimulatory receptor CD2 at the pSMAC of the immune synapse. However, enrichment of the TCR/CD3 complex in the cSMAC remains unchanged 53. In conclusion, activation of the actin-remodeling protein cofilin represents a long-sought molecular mechanism that links T-cell costimulation to actin dynamics-dependent maturation of the immune synapse 50–53.

### Nuclear functions of cofilin

In addition to its function in the cytoplasm, dephosphorylated cofilin has the ability to translocate into the nucleus. Initially, cofilin was detected in intranuclear actin rods following treatment of the mouse fibroblast cell line C3H-ZK with dimethylsulfoxide or following exposure of these cells to heat shock 67. Note that ‘actin/cofilin rods’ do not bind phalloidin. In 1994, we showed for the first time that cofilin translocation into the nucleus succeeds triggering of a cell surface receptor, namely CD2 stimulation of untransformed human T cells 37. By use of single amino acid point mutations, it could be shown that dephosphorylation of cofilin on serine 3 is required to enable its nuclear translocation 35. Cofilin contains a nuclear localization sequence (KKRKK) similar to the nuclear translocation signal sequence of simian virus 40T antigen 68–69 (*Fig. *2). As cofilin but not actin bears a nuclear localization signal, cofilin acts as an actin transporter into the nucleus 70.

In malignant T-lymphoma cells, dephosphorylation of cofilin as well as the nuclear translocation of cofilin together with actin happens spontaneously 71. As shown by confocal laser scanning microscopy following intracellular staining of T-lymphoma cells with rabbit antisera against actin and cofilin, both proteins colocalize within nuclei. Yet, actin/cofilin rods do not occur. The serine phosphatase inhibitor okadaic acid prevents dephosphorylation of cofilin and nuclear translocation of cofilin and actin. Okadaic acid represents an inhibitor of the serine phosphatases PP1 (protein phosphatase 1) and PP2A. These findings imply a role of PP1 or PP2A in the spontaneous dephosphorylation of cofilin in transformed cells. At the same time, okadaic acid induced apoptosis of lymphoma cells 71. Considering the fact that actin has the capability to inactivate DNase I 72, an enzyme involved in apoptosis 73–74, inhibition of the actin import into the nucleus by prevention of cofilin dephosphorylation may contribute to the apoptotic DNA fragmentation observed following okadaic acid treatment.

Besides the potential anti-apoptotic function of cofilin, we speculated that another function of cofilin may be the enhancement of transcriptional processes 37. This conclusion was based on findings that injection of actin antibodies into oocyte nuclei drastically inhibited the transcriptional activity of RNA polymerase II and led to changes in chromosome morphology 75–76. Later it was shown that in mammalian cells actin plays an important role for the regulation of different nuclear processes like transcription, chromatin remodeling, and nucleocytoplasmic trafficking. Importantly, it has been shown only recently in HeLa cells that cofilin is indeed required for RNA polymerase II transcription elongation 77,78. These potential functions of intranuclear actin/cofilin complexes, enhancement of transcription, and prevention of apoptosis, precisely represent mechanisms that one would expect to occur upon malignant transformation as well as following T-cell costimulation. In conclusion, cofilin-mediated actin remodeling is not only a central integrator of costimulation in the immune synapse but rather may fulfill key functions also within the nucleus by enhancing transcription and preventing apoptosis.

### Signaling cascades involved in cofilin dephosphorylation

In response to costimulation, cofilin activation in untransformed human T cells is initiated via a Ras-MEK/PI3K (Rat sarcoma-mitogen-activated protein kinase kinase/phosphoinositide 3-kinase) signaling cascade 51. This was concluded from the following findings: first, transient expression of cDNA-encoded constitutively active Ras in T cells results in dephosphorylation of cofilin. Second, dephosphorylation of cofilin after T-cell costimulation or expression of constitutively active Ras can be prevented by inhibitors of MEK or PI3K. Final proof that the GTPase Ras plays a central role in the regulation of cofilin dephosphorylation came from experiments where we showed that expression of a dominant negative form of Ras in untransformed human T cells prevents activation of PI3K and dephosphorylation of cofilin after costimulation through CD3/CD28. Initially, these findings were surprising, since so far it was thought that PI3K is a substrate of Ras in all cell types except T cells 80. Yet, in the cells that were used in this study, namely the Jurkat T-lymphoma cell line, the situation is indeed different from untransformed T cells. In Jurkat cells, PKB (protein kinase B)/Akt phosphorylation and cofilin dephosphorylation occur spontaneously in the absence of Ras activation 51.

Also, in untransformed human T cells, dephosphorylation of cofilin can be blocked by the serine phosphatase inhibitor okadaic acid 47. Interestingly, both PP1 and PP2A were found to associate with and to dephosphorylate cofilin 81. In principle, the phosphorylation state of cofilin can be influenced by at least two other serine phosphatases, namely slingshot 82 and chronophin 83. Phosphorylation and thereby inactivation of cofilin is mediated by LimKinase (LimK) and testis-specific kinases (TES kinases) (reviewed in 84). The involvement of PP1 in cofilin regulation could recently be confirmed by knockdown of PP1 in untransformed human T cells 85. The contribution of the other enzymes to the regulation of cofilin in untransformed T cells remains to be analyzed in detail.

### Implication of the Ras-cofilin pathway for T-cell anergy prevention

Recognition of antigen without costimulatory signals induces clonal anergy or apoptosis of T cells, a process enabling peripheral tolerance 44–86. Anergic T cells are tolerant toward their antigen and do not execute effector functions. Notably, they are not able to produce IL-2 in response to secondary antigen exposure. Due to the induction of anergy, potential autoreactive T cells that escaped the selection for central tolerance in the thymus and circulate within the blood stream are not hazardous for the organism.

The molecular mechanisms for the induction of peripheral tolerance are not yet fully understood. There are several lines of evidence that an altered regulation of the actin cytoskeleton is involved in the induction or maintenance of T-cell anergy 87–88. As described above, cofilin dephosphorylation is part of a costimulatory signaling pathway and may therefore link costimulation and anergy prevention via its regulatory functions on actin dynamics. Like cofilin activation, full Ras activation is also a costimulation-dependent event in primary human T cells 52 and in mouse T cells 89. Thus, TCR/CD3 triggering alone provokes only a very weak Ras activation, whereas upon costimulation via CD28, a strong (synergistic) activation of Ras is induced. That Ras is indeed important for anergy prevention was underlined by the following findings: first, activation of Ras is blocked in primary anergic T cells 90, and second, expression of constitutively active Ras in once anergic T cells enables them to produce IL-2 91. Since costimulation-induced cofilin dephosphorylation is mediated via Ras 51, it is tempting to speculate that active Ras mediates T-cell anergy prevention via cofilin activation, thereby leading to an increase in actin dynamics. Consequently, blocking the Ras-cofilin pathway in T cells may serve as a valuable means to enable induction of antigen-specific anergy.

## Chemokine-mediated activation of cofilin during migration of T cells

The actin cytoskeleton fundamentally controls the stop-and-go behavior of T cells. Hence, the force vectors induced by radio symmetric actin dynamics in the immune synapse neutralize each other and induce a stop of T-cell migration, which is important for T-cell activation 66–92. T cells leave their cognate APCs after their symmetry is broken through polar forces induced by asymmetric actin dynamics 66. Chemokines then control the migration of T cells in lymphoid organs, their extravasation, and their movement in inflamed tissues.

Triggering of T cells with chemokines induces cell polarization by forming a lamellipodium at the leading edge and a uropod at the rear. In principle, there are two modes of T-cell migration which are used dependent on the microenvironment. On two-dimensional surfaces as they occur on endothelial cells of vessel walls *in vivo* or in petri dishes coated with integrin-ligands *in vitro*, T cells migrate via short-lived interactions of integrins with their substrates and subsequent oscillatory T-cell shape changes mediated via the actomyosin system. This mode of migration is called amoeboid migration 93–94. The situation changes if T cells reach three-dimensional environments in interstitial space or extracellular matrix within tissues. Here, migration is not dependent on integrins. T cells migrate in three-dimensional environments by adapting their shape and squeezing through narrow gaps within the extracellular matrix. This movement is highly dependent on constant actin dynamics that induce an actin flow toward the cell front 94–101.

Interference with cofilin activation or expression has no significant impact on T-cell migration on two-dimensional substrates, as for example on cell culture plates that are coated with ICAM-1 (intercellular adhesion molecule-1) 101. This result is in line with the fact that actin dynamics are of minor importance for amoeboid migration. Cofilin only becomes relevant for T-cell migration on two-dimensional substrates if G-actin availability is limited (e.g. through latrunculin) or if T cells are forced to migrate via constant actin dynamics (e.g. by inhibiting actomyosin via blebbistatin). Importantly, cofilin-dependent actin dynamics are pivotal in three-dimensional environments, as they occur in tissues and can be mimicked by Matrigel® matrices. By interference with the signaling pathway leading to cofilin dephosphorylation or by a knockdown of cofilin expression, we showed that in the absence of cofilin-driven actin-modulation, migration in three-dimensional environments in terms of directionality, velocity, and euclidean distance is strongly reduced 101. Altogether, this demonstrates that the cofilin-mediated dynamic rearrangement of the actin cytoskeleton is the engine that allows crawling of T cells especially in the three-dimensional environment of tissues.

T-cell migration needs a spatio-temporal regulation of cofilin to assure a timely movement and directionality. In line with the dynamic rearrangement of the actin cytoskeleton at the leading edge, in untransformed human T cells, cofilin is dephosphorylated at the lamellipodium via a Ras-MEK signaling module in response to chemokine stimulation 101. Mizuno and coworkers 102 reported a chemokine-induced phosphorylation rather than a dephosphorylation of cofilin. Differing from our experiments, their data were derived from Jurkat lymphoma cells. In this system, however, the phosphorylation state of cofilin is shifted toward the dephosphorylated protein without any stimulation, due to a constitutively active PI3K pathway 51–103. In untransformed human T cells, activation of the Ras-MEK pathway by SDF1α leads to inhibition of the cofilin kinase LimK, thereby shifting the kinase/phosphatase balance toward the phosphatase 101. Since one of the cofilin phosphatases, namely PP2A, is constitutively active in primary human T cells, dominance of PP2A over LimK activity could mediate cofilin dephosphorylation upon chemokine treatment. Contrary to T-cell costimulation, PI3K is not involved in this chemokine-dependent cofilin dephosphorylation 51–101. The difference in dependence of cofilin dephosphorylation on PI3K signaling may result from costimulation-dependent deactivation of PP2A, since this phosphatase was found to be associated with CD28 104. Costimulation-induced deactivation of PP2A would require activation of another cofilin phosphatase to ensure cofilin dephosphorylation, which is presumably regulated by PI3K.

In summary, exposure of primary human T cells to chemokines (e.g. the CXCR4 ligand SDF1α) induces a Ras-MEK-dependent activation of cofilin in the lamellipodium. The resulting onset of actin dynamics at the cell front is crucial for T-cell migration in three-dimensional tissues yet is dispensable for crawling on two-dimensional substrates.

## Phospholipid regulation of cofilin at the cell membrane

Cofilin activation through dephosphorylation starts within 5 min after costimulation of primary human T cells and takes up to 30 min to reach its pinnacle. The initial rise in F-actin occurs during the first few minutes after costimulation or chemokine treatment and declines within the following 5 min. Thereafter, it stays at a level that is still significantly elevated compared to unstimulated cells 105. The kinetics suggests a faster activation of cofilin than observed by its phophoregulation. Such an immediate control of the actin-depolymerizing activity of cofilin is likely mediated by phospholipid regulation. Thus, one of the actin-binding motifs of cofilin also interacts with phosphatidylinositol 4,5-bisphosphate [PI(4,5)P_2_], however, not with its cleavage products inositol 1,4,5-trisphosphate (IP_3_) and diacylglycerol (DAG) (*Fig. *2). PI(4,5)P_2_ binding to cofilin inhibits its capacity to associate with and depolymerize F-actin 106,107. PLC activation, although controlled by TCR signaling, is boosted by CD28-mediated T-cell costimulation, which leads to an enhanced costimulation-dependent PI(4,5)P_2_ cleavage 109,110. Therefore, we postulated that in response to costimulation, a release of cofilin from PI(4,5)P_2_ inhibition may belong to the earliest events of T-cell activation 37. This assumption is supported by recent work showing that stimulation of carcinoma cells with epidermal growth factor leads to a fast hydrolysis of PI(4,5)P_2_, and as a consequence, dephosphorylated cofilin is rapidly released from the cell membrane 39–114. Aside from this temporal regulation of cofilin, phospholipid regulation also provides spatial control of cofilin activity. Cofilin inhibition by PI(4,5)P_2_ takes place near the cell membrane, whereas phosphorylation (inactivation) of cofilin by LimK is compartmentalized to the cytosol 40–115. It was proposed that this mechanism explains how cofilin is activated specifically within lamellipodia or invadosomes of metastasizing tumor cells to produce the asymmetric force that is necessary for migration of the tumor cells 40–116.

Regulation of cofilin by PI(4,5)P_2_ cleavage represents an intrinsic molecular mechanism for a spatio-temporal induction of actin dynamics in carcinoma cells. Accordingly, it is tempting to speculate that a phospholipase-mediated liberation of dephosphorylated cofilin from PI(4,5)P_2_ upon T-cell costimulation is responsible for the initial actin dynamics that precedes Ras-dependent cofilin dephosphorylation.

## Redox regulation of cofilin and its impact on T-cell-mediated immune responses

The actin-binding and remodeling abilities of cofilin are influenced not only by intrinsic factors like phosphorylation and PI(4,5)P_2_ binding but also by microenvironmental conditions. Surrounding factors modulate cofilin functions independently and thus allow adaptation of immune responses to specific tissues and microenvironmental circumstances. In this regard, our group was the first one to show that cofilin is directly targeted by both oxidation and reduction, leading to cofilin regulation through thiol modifications in human T cells 85,105. Thereby, cofilin serves as molecular sensor that translates changes of the redox microenvironment into cellular functions.

### Oxidation-induced molecular changes of cofilin result in disturbed actin dynamics

Physiologically, a pro-oxidative milieu exists in the gut, which is important to control T-cell activation in a microenvironment in which these cells must tolerate food antigens 118–121. Pathologically, a shift toward oxidative stress occurs during inflammation where activated granulocytes and macrophages release reactive oxygen species (ROS). Similarly, a pro-oxidative micromilieu prevails in certain tumors. Such an accumulation of ROS causes local or general immunosuppression, which is characterized by T-cell hyporesponsiveness 122,123. This malfunction of T-cell-mediated immunity is accompanied by an altered actin cytoskeleton within these cells 105. When exogenous ROS are present, primary human T cells display not only a higher amount of steady-state F-actin but also have deficiencies in actin modulation upon cell surface receptor triggering. We showed that these disturbed actin dynamics can be explained by a direct oxidation of cofilin: upon oxidation, the structure of cofilin changes, and as a consequence, it loses its actin-depolymerizing ability, even though it is still able to bind F-actin 105. The functional relevance of an observed diminished binding of oxidized cofilin to G-actin remains as yet unknown 125.

Regarding the impaired actin-depolymerizing activity of oxidized cofilin, initially it seemed likely that oxidative stress leads to enhanced cofilin phosphorylation, thereby preventing its activity. Surprisingly, oxidative stress does not promote phosphorylation of cofilin but rather increases the proportion of unphosphorylated cofilin induced by costimulation of T cells 105. Even culturing T cells with hydrogen peroxide (H_2_O_2_) alone is sufficient to induce cofilin dephosphorylation over time without further stimuli. As described above, MEK and PI3K (downstream effectors of Ras) are key proteins in the signaling cascade leading to cofilin dephosphorylation after T-cell costimulation 51. Although these two proteins are activated by oxidative stress (yet independently of Ras), they do not play a role in the H_2_O_2_-induced diminished phosphorylation of cofilin 105. Thus, the molecular mechanisms propagating decreased cofilin phosphorylation after T-cell costimulation versus oxidative stress are distinct from each other. Our studies revealed that oxidized cofilin is a poor target for the cofilin kinase LimK, which leads to an increase in the proportion of unphosphorylated yet inactive cofilin 105.

Thiol groups of cysteines are the most prominent targets for oxidation in proteins. They can form reversible inter- or intramolecular disulfide bonds or can be modified into higher oxidized (in part irreversible) forms like sulfinic (RSO_2_H) or sulfonic (RSO_3_H) acids 126. Cofilin contains four cysteine residues (at the positions 39, 80, 140, and 148) that are potential targets for ROS (*Fig. *2). Indeed, H_2_O_2_ treatment induces a slight mobility shift of cofilin in non-reducing SDS-PAGE. Addition of the reducing agent dithiothreitol abolishes this shift, suggesting reversible structural changes and the presence of at least one disulfide bridge in oxidized cofilin 105. In the tertiary structure of cofilin, cysteines 39 and 80 are buried inside the molecule, whereas cysteines 139 and 147 face the outside 127. By electrospray mass spectrometry of human recombinant cofilin, we revealed that under oxidative stress conditions, cysteine 139 is modified to a sulfonic acid (Cys-SO_3_H), and the inner cysteines 39 and 80 are likely engaged in an intramolecular disulfide bridge 105 (*Fig. *2). Disulfide bridge formation within the cofilin molecule was later independently confirmed in COS cells and B-lymphoma cells treated with taurine chloramine (TnCl), another oxidizing agent 125. In addition, the existence of another intramolecular disulfide bridge between cysteines 139 and 147 was proposed in that study. Moreover, intermolecular disulfide bridges leading to cofilin dimers or oligomers have been described *in vitro* 128 and in glutamate-stressed neuronal cells 129. Altogether, cofilin oxidation is a phenomenon that has been described in different cell types and under diverse oxidative conditions; however, the susceptibility toward oxidative stress and the functional consequences differ between cell types.

### Cofilin cysteine-oxidation leads to T-cell hyporesponsiveness

As described above, oxidative stress leads to an impaired actin remodeling by cofilin. On the cellular level, impaired actin dynamics during antigen-specific T-cell activation in the presence of H_2_O_2_ manifest in a disturbed immune synapse maturation: cofilin as well as LFA-1 do not properly localize to the T cell/APC contact zone 105, which is reminiscent of the phenotype observed if the interaction of cofilin with actin is blocked by cell-permeable cofilin peptide homologues 53. In accordance with its effects on immune synapse formation, oxidative stress interferes with T-cell activation and consequently induces T-cell hyporesponsiveness 105. This hyporesponsive state is reinforced by inhibition of the migratory capacity of T cells in a pro-oxidative microenvironment. Hence, T cells that are exposed to H_2_O_2_ exhibit defective F-actin polarization, are not able to adhere to immobilized ICAM-1, and lose their ability to migrate toward chemokine gradients. Interestingly, T cells whose endogenous cofilin is replaced by C39G or C80G cofilin (carrying a glycine instead of a cysteine at position 39 or 80) show similar characteristics as oxidatively stressed T cells (e.g. costimulation-dependent activation is strongly impaired if T cells express C39G cofilin even in the absence of ROS). The cysteine-to-glycine mutants C39G and C80G cofilin are therefore on the functional level considered as oxidation-mimicking cofilin mutants 105–117.

### Mitochondrial translocation of oxidized cofilin provokes necrotic-like programmed cell death

Exposing primary human T cells to H_2_O_2_ for expanded time periods can even result in T-cell death 117,130. Electron microscopy and InFlow microscopy revealed that cofilin itself localizes within mitochondria of T cells after long-term treatment with H_2_O_2_ 117. Using our oxidation-mimicking cofilin mutants, we identified the oxidation-induced changes of cofilin as the relevant molecular switch that targets cofilin to the mitochondria. A single point mutation at cysteine 39 or 80 to glycine is sufficient for mitochondrial translocation of cofilin and induction of T-cell death. A change of cysteine to non-oxidizable alanine in cofilin, in contrast, can rescue T cells from death induced by H_2_O_2_. These two types of cofilin mutants, oxidation-mimicking cysteine-to-glycine mutants and oxidation-resistant cysteine-to-alanine mutants, are valuable tools to further elucidate the molecular and cellular mechanisms leading to and resulting from cofilin oxidation.

Chua and colleagues 132 demonstrated that cofilin translocates to mitochondria in staurosporine-treated mammalian cell lines (e.g. COS-7) and that this cofilin translocation is an early step in cell death. The molecular mechanism of cofilin transport to the mitochondria, however, was not elucidated in this study. In T cells, heat-shock cognate protein 70 (HSC70), a chaperone transporting certain proteins into mitochondria 133–134, seems to be one effector molecule in transferring oxidized cofilin to the mitochondria. Upon long-term oxidative stress, HCS70 binds to and colocalizes with cofilin at the mitochondrial membrane 117. Moreover, the oxidation-mimicking cofilin mutants bind to HSC70 even in the absence of H_2_O_2_. Having discovered that cofilin translocation into mitochondria is controlled by oxidation of cofilin and a guided transport mechanism, it seemed likely that cofilin is directly involved in the induction of T-cell death upon oxidative stress. In line with this assumption, targeting of cofilin to mitochondria by introducing the oxidation-mimicking mutants into T cells 117 or targeting of cofilin into mitochondria of COS cells by fusion to a mitochondrial targeting sequence 132 leads to mitochondrial disintegration and cytochrome C release. Despite this cytochrome C release, T-cell death provoked by treatment with H_2_O_2_ or by expression of oxidation-mimicking cofilin mutants is caspase independent 117. In addition, no DNA fragmentation or other signs of apoptosis (or autophagy) are detectable. These observations define the H_2_O_2_-induced T-cell death to be of a necrotic-like phenotype. The fact that expressing oxidation-resistant cofilin mutants or downregulation of cofilin via siRNA rescues the majority of T cells from oxidant-induced cell death highlights the indispensable role of cofilin in the H_2_O_2_-triggered necrotic-like T-cell death and demonstrates that it is a programmed rather than an accidental type of cell death 117–135. The sensitivity toward oxidative stress depends on the T-cell subpopulation. Naive T cells are less sensitive toward oxidative stress induced by low micromolar concentrations of H_2_O_2_ than central and effector memory cells 130. Moreover, the mode of cell death seems to be dependent on the cell death-inducing agent and cell type analyzed. Thus, in contrast to H_2_O_2_-induced necrotic-like programmed cell death in untransformed human T cells, staurosporine-induced and cofilin-dependent cell death of COS cells 132 as well as TnCl-induced cell death in COS cells and B-lymphoma cells 125 were reported to occur by apoptosis rather than necrotic-like programmed cell death.

### Reduction of cofilin as a spatio-microenvironmental control mechanism of actin dynamics

As described above, accumulation of ROS can have detrimental effects on T-cell functions and survival 105–117. Without effective protection, T cells would malfunction in every situation in which a pro-oxidative milieu predominates. To overcome this problem, the body has evolved mechanisms to guard T cells from such an oxidative stress-mediated immune suppression. Dendritic cells (DCs), for example, can induce the upregulation of free thiols inside antigen-specific T cells during an infection and can thereby rescue them from harmful consequences of ROS at the site of inflammation 136,137. Which proteins are affected by this environment, however, remained unclear. Recently, we found that cofilin is such a target for reduction; treatment of human cofilin with reducing agents results in changes of its 3D-(NMR)-structure 85. These structural changes do not alter the actin-depolymerizing function of cofilin in the absence of PI(4,5)P_2_. However, they render dephosphorylated cofilin insensitive toward inhibition by PI(4,5)P_2_ [although PI(4,5)P_2_ still binds to reduced cofilin]. Thus, under reducing conditions, an additional pool of active cofilin, which is still anchored to the membrane by PI(4,5)P_2_, can lead to increased actin dynamics especially during immune synapse formation. In line with this observation, we found that in untransformed human T cells the F-actin content in the immune synapse is diminished, if the T cell/APC contact occurs within a reducing milieu.

In conclusion, reduction of cofilin, which renders it insensitive to PI(4,5)P_2_ inhibition, may raise the amounts of active cofilin near the plasma membrane. Thereby, a reducing milieu may upregulate actin dynamics at the immune synapse and the rate of T-cell activation. In a pro-oxidative environment, cofilin reduction may enhance resistance toward oxidative stress-induced T-cell hyporesponsiveness. Ultimately, for the fate of the T cell, it may be crucial which side of the redox spectrum outweighs the other, for example, how long the protective reducing intracellular milieu is exposed to oxidative stress.

### Comprehensive model of spatio-temporal and microenvironmental regulation of cofilin

**Figure 4:**
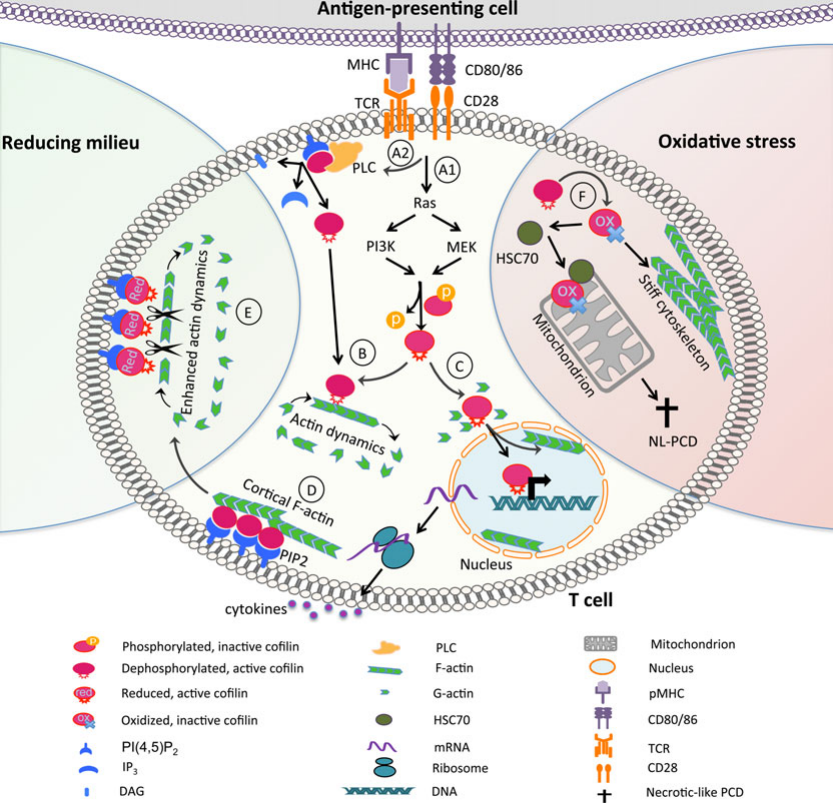
Spatio-temporal and microenvironmental control of cofilin in T cells. Costimulation induces cofilin activation via Ras (A1), which results in cofilin dephosphorylation in the cytoplasm, and via PLC (A2), which liberates dephosphorylated cofilin from PI(4,5)P_2_ inhibition. This results in the onset of activation-induced actin dynamics (B). In addition to its functions for actin dynamics, dephosphorylated cofilin can carry actin into the nucleus (C). Thereby, it can modulate gene transcription by altering the nuclear actin pool and the activity of RNA polymerase II. PI(4,5)P_2_ bound cofilin is inactive and detains F-actin at the plasma membrane (= cortical actin, D). In the presence of a reducing milieu, this cofilin pool gets active despite binding to PI(4,5)P_2_. Thereby, actin dynamics near the plasma membrane are enhanced (E). In contrast, a strong pro-oxidative milieu can oxidize (inactivate) cofilin which results in a stiff actin cytoskeleton and T-cell hyporesponsiveness or even necrotic-like programmed cell death (NL-PCD) through mitochondrial disintegration (F).

In this review, we have described that cofilin-induced actin dynamics are important for immune synapse formation, T-cell activation 50,53, and T-cell migration 101. Taking these vital functions of cofilin into account, it is clear that cofilin needs to be tightly controlled in different subcellular compartments. Here, we introduce a model of ‘spatio-temporal and microenvironmental control of cofilin in T cells’ (*Fig. *4). It is based on *in vitro* data and data derived from T cells or other cellular systems. In resting human T cells, cofilin is mainly inactive and exists in distinct subcellular locations. Cytoplasmic cofilin is mainly phosphorylated and thus in an inactive state. The membrane-bound fraction of cofilin is dephosphorylated but kept inactive by binding to PI(4,5)P_2_. Both the cytoplasmic fraction and at least a proportion of the membrane-bound cofilin are activated by T-cell costimulation. Cytoplasmic cofilin becomes dephosphorylated through costimulation-induced activation of Ras and its downstream effectors PI3K and MEK 51. Membrane-bound dephosphorylated cofilin can be activated by PLC-dependent PI(4,5)P_2_ cleavage releasing dephosphorylated cofilin into the cytoplasm 38,40. Thereby, the cytoplasmic pool of activated cofilin is increased and actin dynamics are reinforced. Moreover, dephosphorylated cofilin can translocate into the nucleus 37, where it may act as actin shuttle and as chaperone for RNA polymerase II-dependent gene transcription 70–141.

Although costimulation boosts PLC activation, a large amount of PI(4,5)P_2_ remains uncleaved. Therefore, a significant fraction of cofilin remains inactive at the plasma membrane. This PI(4,5)P_2_-bound fraction can be activated *in situ* by the creation of a reducing milieu in T cells. Thus, antigen-specific T-cell activation via DCs not only provides costimulation but DCs can also induce an increase of free thiols within T cells in an antigen-specific manner 136. Such a reducing milieu changes the structure of cofilin and makes it insensitive toward inhibition by PI(4,5)P_2_ 85. The resulting additional activation of cofilin at the plasma membrane in a reducing environment does not only increase the total pool of active cofilin within T cells but also represents a spatial control of cofilin function. This mechanism appears to be especially important for the dynamics of cortical actin as the activated cofilin is still bound to PI(4,5)P_2_ and thus remains at the plasma membrane.

The reducing conditions induced by DCs 136 can also protect T cells surrounded by a pro-oxidative environment. Oxidative stress occurs if ROS exceed the reducing potential within the cytoplasm. Cofilin becomes inactivated during oxidative stress, which ultimately leads to T-cell hyporesponsiveness due to an inability of cofilin to induce actin dynamics and eventually a stiffening of the actin cytoskeleton 105. Moreover, as a long-term effect, oxidized cofilin binds to HSC70 and translocates into the mitochondria, provoking their disintegration and induction of necrotic-like programmed T-cell death 117. Altogether, signal transduction pathways, factors of the microenvironment, or both can control each of these cofilin pools in different subcellular localizations with distinct kinetics, thereby influencing the outcome of a T-cell-mediated immune response.

## Relevance of cofilin in diseases

### The role of cofilin for uptake and spreading of pathogens

Cortical F-actin represents a mechanical barrier for entry of pathogens into T cells. Thus, pathogens need to induce phagocytosis and/or an active breakdown of the F-actin structures near the plasma membrane to get access to the host cell. Since cofilin represents the main actin-depolymerizing factor, some pathogens like *Listeria monocytogenes* and the human immunodeficiency virus (HIV) evolved mechanisms to highjack cofilin to increase their pathogenicity.

Listeriosis is an infectious disease caused by the Gram-positive bacterium *Listeria monocytogenes*. The severity of the disease is in part due to the ability of *L. monocytogenes* to evade the immune system by hiding within the cytoplasm of host cells. *L. monocytogenes* can not only persist within the host cells but also spread through cell-to-cell migration without reaching the extracellular space (reviewed in 142). The cell invasion and cell-to-cell spreading is in different steps dependent on cofilin, and, thus, cofilin is an important factor in the pathology of listeriosis. The surface receptor internalin B of *L. monocytogenes* binds to the MET-receptor and increases the level of phospho-cofilin via activation of LimK in the host cytoplasm. Phosphorylated cofilin is inactive for induction of actin dynamics, but it is necessary for activation of phospholipase D, which is required for the invasion of *L. monocytogenes* into host cells 143–144. However, the inactivation of cofilin must be balanced during *L. monocytogenes* internalization, since, as mentioned above, the barrier of cortical F-actin needs to be broken down through cofilin activation 145. In addition to its role for host cell penetration, *L. monocytogenes* recruits the host's actin cytoskeleton to create actin comet tails within the cytoplasm. Cofilin is one important factor to create actin dynamics in the comet tail to generate a force for bacterial movement within the host cell 146–147. Importantly, this actin-based movement enables *L. monocytogenes* to spread into neighboring cells without reaching the extracellular space. This keeps the bacteria hidden from phagocytosis by innate immune cells and prevents their antibody-mediated opsonization or neutralization. Thus, cofilin regulation is an important step for the pathogenicity of *Listeria* spp. 142–148.

HIV infects human T cells and thus interferes with T-cell-mediated immunity. Yoder and colleagues 149 demonstrated that HIV triggers its uptake by binding to the HIV co-receptor CXCR4. Binding of HIV to CXCR4 activates cofilin, which consequently breaks down the cortical actin through the induction of actin dynamics, allowing entry of the virus. We have recently shown that T-cell stimulation via the chemokine receptor CXCR4 through it natural ligand SDF1α leads to cofilin activation via a Ras-MEK signaling module and an acceleration of actin dynamics 101. It would be interesting to know whether the HIV-induced cofilin dephosphorylation follows the same signaling pathways as triggered by SDF1α to allow virus entry. After the successful infection, the HIV genome is transcribed within the cytoplasm of the host cell. Thereby, the negative regulatory factor (Nef) is expressed as one important protein for HIV pathogenesis. Expression of Nef induces phosphorylation and thus deactivation of cofilin via binding to p21-activated kinase 2 (Pak2) 149–151. It was suggested that this cofilin inactivation is pivotal for inhibiting the chemokine-dependent migration of HIV-infected T cells. Eventually, these T cells are not able to provide B-cell help and thus the induction of germinal centers and production of high affinity antibodies is dampened. In addition, the slowing down of HIV-infected T cells may induce a microenvironment enriched by these cells and thereby facilitate infection of bystander T cells. Thus, HIVs use cofilin at different stages of their infection cycle.

### The role of cofilin in cancer

In malignant T-lymphoma cells the phosphorylation state of cofilin is shifted toward the dephosphorylated and thus active form of cofilin 37. This is a tumor-promoting mechanism, since a cofilin knockdown diminishes the cloning efficiency of these cells and interference with the respective cofilin phosphatases induces apoptosis of T-lymphoma cells 71. Cofilin is also constitutively dephosphorylated in non-hematopoietic tumor cells, such as the cervix carcinoma cell line HeLa or the colon carcinoma cell line KM12 35. Within the last decade, cofilin expression and its activation state were described as determining the metastatic potential of tumor cells. They represent major factors of the severity of the disease in several cancer entities, such as breast cancer 116–152. One main function of cofilin in these tumor cells is its role for chemotaxis, directionality 153–154, and the maturation of the invadopodium 155–156.

Another mechanism favoring cancer progression is the potential downregulation of cofilin activity in T cells by tumor cells or tumor-infiltrating immune cells like macrophages, granulocytes, and myeloid derived suppressor cells (MDSCs). These can produce high amounts of immunosuppressive mediators (e.g. ROS or TGFβ) 123–124. Given that cofilin is a direct target for ROS and oxidized cofilin mediates T-cell hyporesponsiveness or necrotic-like programmed cell death 105–117, this may be detrimental, since adaptive immunity against tumor cells is hindered. Myeloid cells regulate not only the redox milieu but also amino acid catabolism. Arginase I is an enzyme that hydrolyses arginine and induces arginine deprivation in the surrounding tissues. It is constitutively expressed in human neutrophils and is inducibly produced by MDSCs 157–158. The physiological role of arginase I production and release is the balancing of immune responses to avoid excessive tissue destruction during inflammation (e.g. pus inducing infections) 159. Too high arginase I activity can lead, however, to arginine deprivation, thereby provoking T cell hyporesponsiveness, for example, in tumor microenvironments 160,161. We found that such an arginine deprivation in the environment of human T cells increases the amount of phosphorylated, inactive cofilin. Accordingly, immune synapse formation between T cells and APCs and the resulting T-cell activation are inhibited 163. Notably, while polyclonal and antigen-specific proliferation of pan T cells are diminished, the antigen-specific cytotoxicity of CD8^+^ T cells, which occurs through a different type of immune synapse, remains unaffected by arginine deprivation 164.

### Potential role of cofilin in chronic inflammation/autoimmune diseases

Whereas the relevance of cofilin for infectious and neoplastic diseases is without dispute, the evidence for an involvement of cofilin in chronic inflammatory/autoimmune disorders are in fact more indirect but conclusive. Notably, autoantibodies directed against cofilin have been found in patients suffering from rheumatoid arthritis, systemic lupus erythematosus, polymyositis, dermatomyositis, and Behçet's disease 165. This finding implies a diagnostic and/or pathological role of cofilin in autoimmune diseases. Moreover, the environmental control of cofilin activity could be an important key for the pathological onset or propagation of chronic inflammatory diseases. Thus, a knockout of p47phox disturbs activation of NADPH (nicotinamide adenine dinucleotide phosphate) oxidase and thus the oxidative burst in granulocytes. The p47phox knockout in mice or rats increases the severity of T cell-dependent but not of T cell-independent arthritis 166–167. It is tempting to speculate that a shift of the redox state of cofilin toward reduction may be one molecular mechanism that leads to the pathological activation of T cells in the joints of these rodents. Notably, dephosphorylation/activation of cofilin is not blocked by cyclosporine A or FK506, important immunosuppressive drugs targeting the serine phosphatase PP2B (protein phosphatase 2B/calcineurin) 81. Other well-known immunosuppressive drugs for example, mycophenolic acid, leflunomide, dexamethasone, and rapamycin, also do not influence this signaling event. Therefore, enzymes regulating the activity of cofilin (either directly or by influencing the microenvironment) may represent interesting novel targets for therapeutic immune modulation.

## Concluding remarks

**Figure 5:**
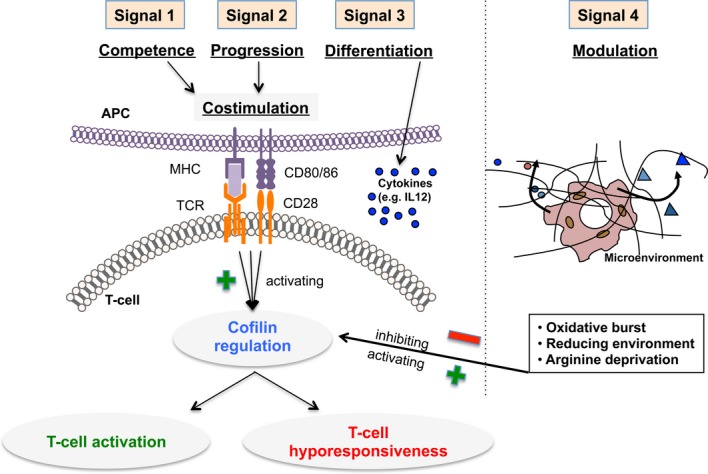
The ‘four signal model of T-cell activation’. T-cell costimulation via the TCR/CD3 complex (competence or first signal) and costimulatory receptors (progression or second signal) is mandatory to induce cofilin dephosphorylation and full T-cell activation. Neither the first nor second signal alone is sufficient to provoke cofilin and T-cell activation. The third signal mediates T-cell differentiation via cytokines like IL-12. Effects on cofilin are as yet unknown. A fourth signal is provided by the microenvironment. This fourth signal has a modulating function that adapts T-cell responses to the conditions in the surrounding tissue. It can be activating (e.g. reducing milieu) or inhibiting (e.g. oxidative milieu or arginine deprivation).

The widespread relevance of cofilin for T-cell activation and migration as well as its implications in different diseases suggest that pharmacological manipulation of the signaling pathways leading to cofilin activation or a cell type-specific inhibition of cofilin expression could provide effective means to improve the outcome of various diseases. Recently, an additional mode of cofilin regulation has been discovered: the redox regulation of cofilin 85,105. It is independent of receptor triggering and cytokine functions and thus provides a previously unrecognized microenvironmental signal influencing T-cell-mediated immune responses. Therefore, we propose a ‘four signal model for T-cell activation’ (*Fig. *5). The classical competence signal (first signal) of T-cell activation is provided by the peptide-MHC-complex (pMHC) and its recognition through the antigen-specific TCR/CD3-complex. A second signal through costimulation of accessory receptors (e.g. CD28 or CD2) is essential to avoid TCR-induced anergy. It induces cofilin dephosphorylation 37 and full T-cell activation 44,168. The third signal is elicited through particular cytokine combinations, which are needed for the differentiation, development, and polarization of helper T cells (e.g. Th1, Th2, or Th17) or cytotoxic T cells (reviewed in 170–171). The microenvironmental regulation of T cells can be described, as a logical extension of this concept, as a fourth or modulating signal for T-cell activation and function. Cofilin is a key molecule that senses alterations in the redox microenvironment as well as arginine deprivation and translates these signals into T-cell functions via its impact on actin dynamics. Notably, this fourth signal of T-cell activation and modulation is not only restricted to the activation of naive T cells in the lymphoid organs but also represents a modulatory mechanism for T-cell-mediated immunity of naive and memory T cells in non-lymphoid organs and inflamed tissues. Future research should take this modulating fourth signal more into account to fully understand tissue-specific immune responses.
